# Complex temporal topic evolution modelling using the Kullback-Leibler divergence and the Bhattacharyya distance

**DOI:** 10.1186/s13637-016-0050-0

**Published:** 2016-09-29

**Authors:** Victor Andrei, Ognjen Arandjelović

**Affiliations:** School of Computer Science, University of St Andrews, St Andrews KY16 9SX, Fife, Scotland, UK

**Keywords:** Topic modelling, Dirichlet process, Bayesian, Temporal graph, Hierarchical model

## Abstract

The rapidly expanding corpus of medical research literature presents major challenges in the understanding of previous work, the extraction of maximum information from collected data, and the identification of promising research directions. We present a case for the use of advanced machine learning techniques as an aide in this task and introduce a novel methodology that is shown to be capable of extracting meaningful information from large longitudinal corpora and of tracking complex temporal changes within it. Our framework is based on (i) the discretization of time into epochs, (ii) epoch-wise topic discovery using a hierarchical Dirichlet process-based model, and (iii) a temporal similarity graph which allows for the modelling of complex topic changes. More specifically, this is the first work that discusses and distinguishes between two groups of particularly challenging topic evolution phenomena: topic splitting and speciation and topic convergence and merging, in addition to the more widely recognized emergence and disappearance and gradual evolution. The proposed framework is evaluated on a public medical literature corpus.

## Introduction

Recent years have witnessed a remarkable convergence of two broad trends. The first of these concerns information, i.e. data, rapid technological advances coupled with an increased presence of computing in nearly every aspect of daily life, have for the first time made it possible to acquire and store massive amounts of highly diverse types of information. Concurrently and in no small part propelled by the environment just described, research in artificial intelligence—in machine learning [3–5, 8], data mining [14], and pattern recognition, in particular—has reached a sufficient level of methodological sophistication and maturity to process and analyse the collected data, with the aim of extracting novel and useful knowledge [7, 14–16]. Though it is undeniably wise to refrain from overly ambitious predictions regarding the type of knowledge which may be discovered in this manner, at the very least, it is true that few domains of application of the aforesaid techniques hold as much promise and potential as that of medicine and health in general.

Large amounts of highly heterogeneous data types are pervasive in medicine. Usually the concept of so-called big data in medicine is associated with the analysis of Electronic Health Records [6, 7, 9, 21, 40, 41, 43], large scale sociodemographic surveys of death causes [37], clinical epidemiological [17, 23, 36] and pharmacoepidemiological studies [28, 35, 42], as well as in the analysis of pharmacovigilance [22, 29, 32], health-related economic effects [11, 20], and public health [18, 27, 30, 34]. Much less discussed and yet arguably no less important realm where the amount of information presents a challenge to the medical field is the medical literature corpus itself. Namely, considering the overarching and global importance of health (to say nothing of practical considerations such as the availability of funding), it is not surprising to observe that the amount of published medical research is immense and its growth is only continuing to accelerate. This presents a clear challenge to a researcher. Even restricted to a specified field of research, the amount of published data and findings makes it impossible for a human to survey the entirety of relevant publications exhaustively which inherently leads to the question as to what kind of important information or insight may go unnoticed or insufficiently appreciated. The premise of the present work is that advanced machine learning techniques can be used to assist a human in the analysis of this data. Specifically, we introduce a novel algorithm based on Bayesian non-parametric inference that achieves this goal.

### Previous work

A limitation of most models described in the existing literature lies in their assumption that the data corpus is static. Here the term ‘static’ is used to describe the lack of any associated temporal information associated with the documents in a corpus—the documents are said to be exchangeable [19]. However, research articles are added to the literature corpus in a temporal manner and their ordering has significance. Consequently, the topic structure of the corpus changes over time [12, 13, 24]: new ideas emerge, old ideas are refined, novel discoveries result in multiple ideas being related to one another thereby forming more complex concepts or a single idea multifurcating into different ‘sub-ideas’, etc. The premise in the present work which expands on our preliminary contributions described in [1, 2] is that documents are not exchangeable at large temporal scales but can be considered to be at short time scales, thus allowing the corpus to be treated as *temporally locally static*.

## Methods

In this section, we introduce our main technical contributions. We begin by reviewing the relevant theory underlying Bayesian mixture models and then explain how the proposed framework employs these for the extraction of information from temporally varying document corpora.

### Bayesian mixture models

Mixture models are appropriate choices for the modelling of the so-called heterogeneous data whereby heterogeneity is taken to mean that observable data is generated by more than one process (source). The key challenges lie in the lack of observability of the correspondence between specific data points and their sources and the lack of a priori information on the number of sources [38].

Bayesian non-parametric methods place priors on the infinite-dimensional space of probability distributions and provide an elegant solution to the aforementioned modelling problems. Dirichlet Process (DP) in particular allows for the model to accommodate a potentially infinite number of mixture components [25]:

1 $$\begin{array}{*{20}l} p\left(x|\pi_{1:\infty},\phi_{1:\infty}\right)=\sum_{k=1}^{\infty}\pi_{k}f\left(x|\phi_{k}\right). \end{array} $$

where DP(*γ*,*H*) is defined as a distribution of a random probability measure *G* over a measurable space $\left (\Theta,\mathcal {B}\right)$, such that for any finite measurable partition (*A*_1_,*A*_2_,…,*A*_*r*_) of *Θ* the random vector (*G*(*A*_1_),…,*G*(*A*_*r*_)) is a Dirichlet distribution with parameters (*γ* *H*(*A*_1_),…,*γ* *H*(*A*_*r*_)). A Dirichlet process mixture model (DPM) is obtained by associating different mixture components with atoms *ϕ*_*k*_ and assuming $x_{i}|\phi _{k}\overset {iid}{\sim }f\left (x_{i}|\phi _{k}\right)$ where *f*(.) is the kernel of the mixing components [33].

#### Hierarchical DPMs

While the DPM is suitable for the clustering of exchangeable data in a single group, many real-world problems are more appropriately modelled as comprising multiple groups of exchangeable data. In such cases, it is desirable to model the observations of different groups jointly, allowing them to share their generative clusters. This ‘sharing of statistical strength’ emerges naturally when a hierarchical structure is implemented.

The DPM models each group of documents in a collection using an infinite number of topics. However, it is desirable for multiple group-level DPMs to share their clusters. The hierarchical DP (HDP) [39] offers a solution whereby base measures of group-level DPs are drawn from a corpus-level DP. In this way, the atoms of the corpus-level DP are shared across the documents; posterior inference is readily achieved using Gibbs sampling [39].

### Modelling topic evolution over time

We now show how the described HDP based model can be applied to the analysis of temporal topic changes in a *longitudinal* data corpus.

Owing to the aforementioned assumption of a temporally locally static corpus, we begin by discretizing time and dividing the corpus into epochs. Each epoch spans a certain contiguous time period and has associated with it all documents with timestamps within this period. Each epoch is then modelled separately using a HDP, with models corresponding to different epochs sharing their hyperparameters and the corpus-level base measure. Hence if *n* is the number of epochs, we obtain *n* sets of topics $\boldsymbol {\phi }=\left \{ \boldsymbol {\phi }_{t_{1}},\ldots,\boldsymbol {\phi }_{t_{n}}\right \}$ where $\boldsymbol {\phi }_{t}=\left \{ \phi _{1,t},\ldots,\phi _{K_{t},t}\right \} $ is the set of topics that describe epoch *t*, and *K*_*t*_ their number.

#### Topic relatedness

Our goal now is to track changes in the topical structure of a data corpus over time. The simplest changes of interest include the emergence of new topics and the disappearance of others. More subtly, we are also interested in how a specific topic changes, that is, how it evolves over time in terms of the contributions of different words it comprises. Lastly, our aim is to be able to extract and model complex structural changes of the underlying topic content which result from the interaction of topics. Specifically, topics, which can be thought of as collections of memes, can merge to form new topics or indeed split into more nuanced memetic collections. This information can provide valuable insight into the refinement of ideas and findings in the scientific community, effected by new research and accumulating evidence.

The key idea behind our tracking of simple topic evolution stems from the observation that while topics may change significantly over time, changes between successive epochs are limited. Therefore, we infer the continuity of a topic in one epoch by relating it to all topics in the immediately subsequent epoch which are sufficiently similar to it under a suitable similarity measure—we adopt the well-known Bhattacharyya distance (BHD):

2 $$\begin{array}{*{20}l} \rho_{\text{BHD}}(p,q) = -\ln \sum_{i} \sqrt{p(i) q(i)} \end{array} $$

where *p*(*i*) and *q*(*i*) are two probability distributions. This approach can be seen to lead naturally to a similarity graph representation whose nodes correspond to topics and whose edges link those topics in two epochs which are related. Formally, the weight of the directed edge that links *ϕ*_*j*,*t*_, the *j*th topic in epoch *t*, and *ϕ*_*k*,*t*+1_ is *ρ*_BHD_(*ϕ*_*j*,*t*_,*ϕ*_*k*,*t*+1_) where *ρ*_BHD_ denotes the BHD.

In constructing a similarity graph a threshold is used to eliminate automatically weak edges, retaining only the connections between sufficiently similar topics in adjacent epochs. Then, the disappearance of a particular topic, the emergence of new topics, and gradual topic evolution can be determined from the structure of the graph. In particular, if a node does not have any edges incident to it, the corresponding topic is taken as having emerged in the associated epoch. Similarly, if no edges originate from a node, the corresponding topic is taken to vanish in the associated epoch. Lastly, when exactly one edge originates from a node in one epoch and it is the only edge incident to a node in the following epoch, the topic is understood as having evolved in the sense that its memetic content may have changed.

**Fig. 1:**
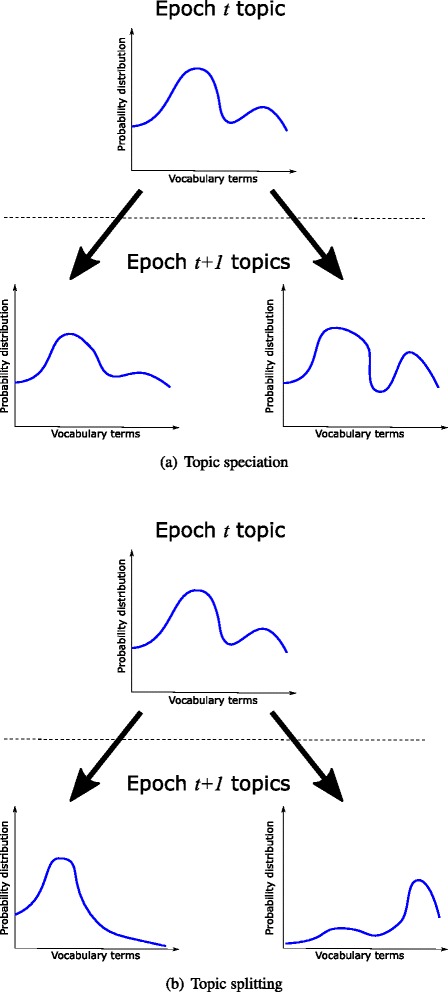
We are the first to recognize and describe the difference between two topic evolution phenomena: **a** topic speciation and **b** topic splitting

A major challenge to the existing methods in the literature concerns the detection of topic merging and splitting. Since the connectedness of topics across epochs is based on their similarity, what the previous work describes as ‘splitting’ or indeed ‘merging’ does not adequately capture these phenomena. Rather, adopting the terminology from biological evolution, a more accurate description would be ‘speciation’ and ‘convergence’, respectively. The former is illustrated in Fig. 1a whereas the latter is entirely analogous with the time arrow reversed. What the conceptual diagram shown illustrates is a slow differentiation of two topics which originate from the same ‘parent’. Actual topic splitting, which does not have a biological equivalent in evolution, and which is conceptually illustrated in Fig. 1b, cannot be inferred by measuring topic similarity. Instead, in this work, we propose to employ the Kullback-Leibler divergence (KLD) for this purpose. The divergence *ρ*_KLD_(*p*,*q*) is asymmetric, and it measures the amount of information lost in the approximation of the probability distribution *p*(*i*) with *q*(*i*). KLD is defined as follows:

3 $$\begin{array}{*{20}l} \rho_{\text{KLD}}(p,q) = \sum_{i} p(i) \ln \frac{p(i)}{q(i)} \end{array} $$

It can be seen that a high penalty is incurred when *p*(*i*) is significant and *q*(*i*) is low. Hence, we use the BHD to track gradual topic evolution, speciation, and convergence, while the KLD (computed both in forward and backward directions) is used to detect topic splitting and merging.

#### Automatic temporal relatedness graph construction

Another novelty of the work first described in this paper concerns the building of the temporal relatedness graph. We achieve this almost entirely automatically, requiring only one free parameter to be set by the user. Moreover, the meaning of the parameter is readily interpretable and understood by a non-expert, making our approach highly usable.

Our methodology comprises two stages. Firstly, we consider all inter-topic connections present in the initial fully connected graph and extract the empirical estimate of the corresponding cumulative density function (CDF). Then, we prune the graph based on the operating point on the relevant CDF. In other words, if *F*_*ρ*_ is the CDF corresponding to a specific initial, fully connected graph formed using a particular similarity measure (BHD or KLD), and *ζ*∈[0,1] the CDF operating point, we prune the edge between topics *ϕ*_*j*,*t*_ and *ϕ*_*k*,*t*+1_ iff $\rho (\phi _{j,t},\phi _{k,t+1}) < F^{-1}_{\rho } (\zeta)$.

## Evaluation and discussion

We now analyse the performance of the proposed framework empirically on a large real-world data set.

### Evaluation data

We used the PubMed interface to access the US National Library of Medicine and retrieve from it scholarly articles. We searched for publication on the autism spectrum disorder (ASD) and the metabolic syndrome (MetS) using respectively the keyword ’autism’ and the keyphrase ‘metabolic syndrome’ and collected only those achieved papers which were written in English. The earliest publications found were that by Kanner [26] on ASD and by Berardinelli et al. [10] on MetS. We collected all matching publications up to the final one indexed by PubMed on 10 May 2015, yielding a corpus of 22,508 publications on ASD and of 31,706 on MetS. We used the corresponding abstracts to evaluate our contributions.

#### Pre-processing

The raw data collected from PubMed is in the form of free text. To prepare it for automatic analysis a series of ‘pre-processing’ steps are required. The goal is to remove words which are largely uninformative, reduce dispersal of semantically equivalent terms, and thereafter select terms which are included in the vocabulary over which topics are learnt.

We firstly applied soft lemmatization using the WordNet®; lexicon [31] to normalize for word inflections. No stemming was performed to avoid semantic distortion often effected by heuristic rules used by stemming algorithms. After lemmatization and the removal of so-called stop-words, we obtained approximately 2.2 and 3.8 million terms in the entire corpus when repetitions are counted and 37,626 and 46,114 unique terms, for the ASD and MetS corpora, respectively. Constructing the vocabulary for our method by selecting the most frequent terms which explain 90 % of the energy in a specific corpus resulted in ASD and MetS vocabularies containing 3417 and 2839 terms, respectively.

### Results and discussion

**Fig. 2:**
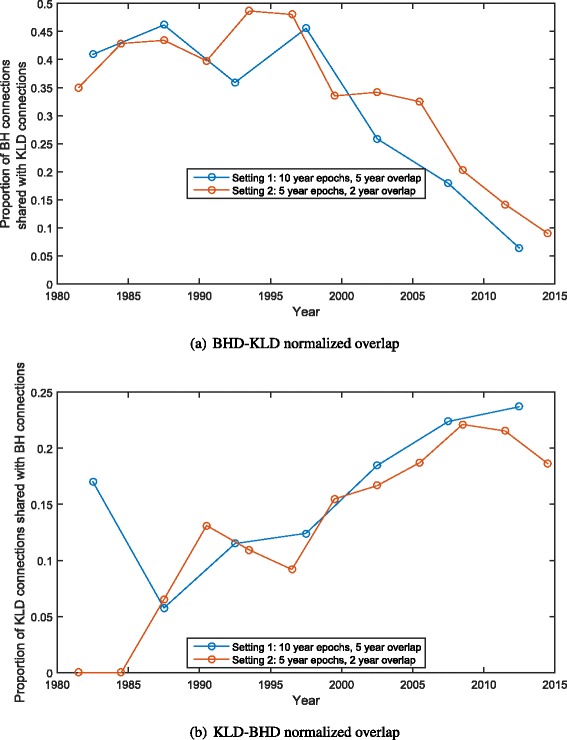
The proportion of topic connections shared between the BHD and the KLD temporal relatedness graphs, normalized by **a** the number of BHD connections and **b** the number of KLD connections, in an epoch. The low overlap of inferred connections demonstrates that the BHD and the KLD indeed do capture different types of topic relatedness

**Fig. 3:**
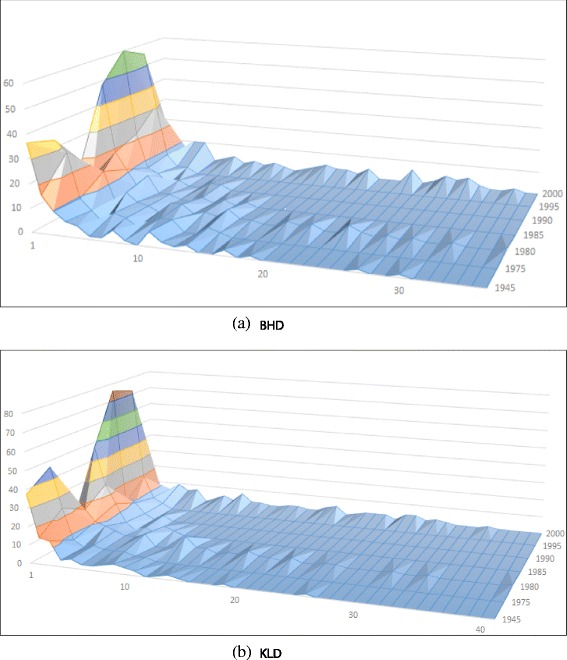
The distributions of the number of connections leading from topics in our topic relatedness graph inferred using the **a** BHD and the **b** KLD using the CDF pruning threshold of *ζ*=0.9. The axis *x*, *y*, and *z* respectively correspond to time (i.e. epochs), number of branches leading from a topic, and the corresponding number of topics

We stared evaluation by examining whether the two topic relatedness measures (BHD and KLD) are capturing different aspects of relatedness. To obtain a quantitative measure, we looked at the number of inter-topic connections formed in respective graphs both when the BHD is used as well as when the KLD is applied instead. The results were normalized by the total number of connections formed between two epochs, to account for changes in the total number of topics across time. Our results for the MetS corpus are summarized in Fig. 2; similar results were obtained for ASD data. A significant difference between the two graphs is readily evident; across the entire timespan of the data corpus, the number of Bhattacharyya distance-based connections also formed through the use of the KLD is less than 40 % and in most cases less than 30 %. An even greater difference is seen when the proportion of the KLD connections is examined—it is always less than 25 % and most of the time less than 15 %. The graphs in Fig. 3 show the distributions of the number of connections leading from topics in our topic relatedness graph inferred using the BHD and the KLD using the CDF pruning threshold of *ζ*=0.9.

**Fig. 4:**
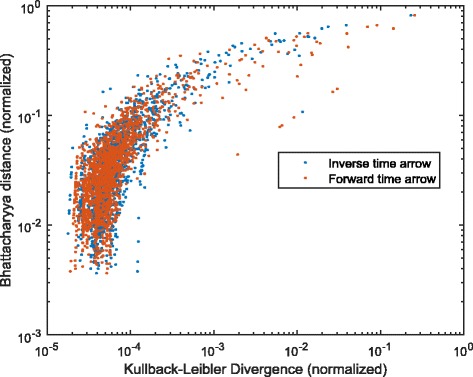
Relationship between inter-topic edge strengths computed using the BHD and the KLD before the pruning of the respective graphs. The high degree of scatter corroborates with an even greater strength our previous observation based on the plots in Fig. 2. In particular, the graph demonstrates that the knowledge of topic relatedness by one of the two distance measures used by our algorithm provides little information on the value of the other distance measure thereby showing that the two capture different types of topic relatedness

To get an even deeper insight into the contribution of the two relatedness measures, we examined the corresponding topic graphs before edge pruning. The plot in Fig. 4 shows the variation in inter-topic edge strengths computed using the BHD and the KLD (in forward and backward directions)—the former as the *x* coordinate of a point corresponding to a pair of topics and the latter as its *y* coordinate. The scatter of data in the plot corroborates our previous observation that the two similarity measures indeed do capture different aspects of topic behaviour.

**Fig. 5:**
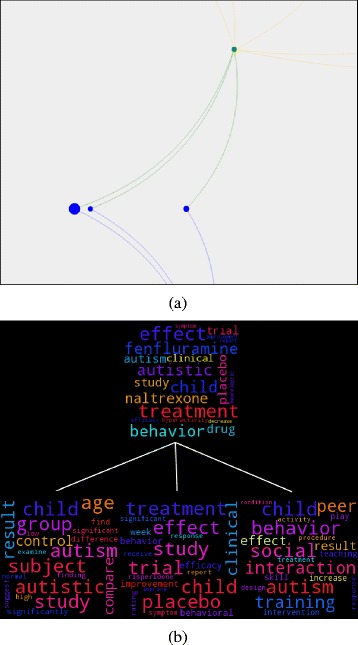
An example of topic splitting as inferred through the use of the KLD by our algorithm but which is undetected by the previously proposed BHD-based relatedness: **a** magnification of the salient window region in our interactive application used to explore large longitudinal text corpora (described in Section 3.3) showing the split topic and its descendant topics and **b** the split topic and its descendent topics shown as word clouds. Recall that the notion of topic splitting as used in this work differs significantly from that used in previous work. As argued in Section 2.2, the previous understanding of topic splitting is better described as topic speciation

**Fig. 6:**
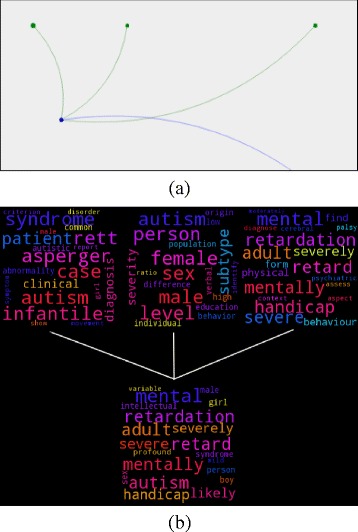
An example of topic merging as inferred through the use of the KLD by our algorithm but which is undetected by the previously proposed BHD-based relatedness: **a** magnification of the salient window region in our interactive application used to explore large longitudinal text corpora (described in Section 3.3) showing the merging topics and their descendant topic and **b** the merging topics and the resultant topic shown as word clouds. Recall that the notion of topic merging as used in this work differs significantly from that used in previous work. As argued in Section 2.2, the previous understanding of topic splitting is better described as topic convergence

We also performed extensive qualitative analysis which is necessitated by the nature of the problem at hand and the so-called semantic gap that underlies it. In all cases, we found that our algorithm revealed meaningful and useful information as verified by medical experts on the ASD and MetS. This observation is consistent with previous reports in the literature using an algorithm with a similar structure [12]. The key novelties which we emphasized in the present article are illustrated well in Figs. 5 and 6, which illustrate respectively topic splitting and topic merging as introduced by us (as opposed to the phenomena of topic speciation and topic convergence, previously incorrectly termed splitting and merging). The splitting and the merging shown are identified through the use of the KLD but not by the BHD based relatedness inference (see Section 2.2).

### Interactive exploration and visualization

**Fig. 7:**
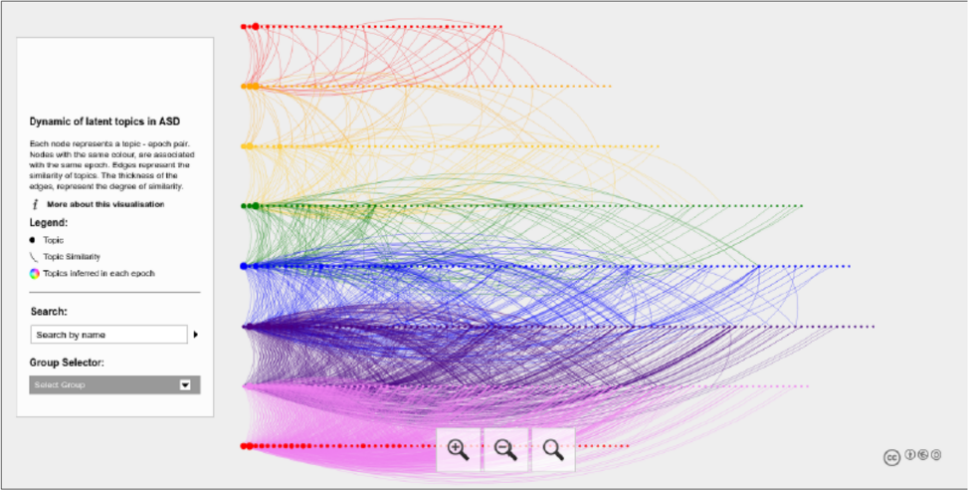
Screenshot of the initial window of the application we developed for free public analysis of custom data sets using the method described in the present paper. Topics are visualized as *coloured blobs* arranged in rows, each row corresponding to a single epoch (with the *time arrow pointing downwards*). The size of each blob is proportional to the popularity of the corresponding topic within its epoch. Each epoch is coloured using a *single colour* different from the neighbouring epochs. *Line connectors between topics* denote temporal topic connections across the temporal relatedness graph and encode topic evolution, merging and splitting, and convergence and speciation

Our final contribution comprises a web application which allows users to upload and analyse their data sets using the proposed framework. A screenshot of the initial window of the application when a data set is loaded is shown in Fig. 7. Topics are visualized as coloured blobs arranged in rows, each row corresponding to a single epoch (with the time arrow pointing downwards). The size of each blob is proportional to the popularity of the corresponding topic within its epoch. Each epoch (that is to say, the set of topics associated with a single epoch) is coloured using a single colour different from the neighbouring epochs for easier visualization and navigation. Line connectors between topics denote temporal topic connections, i.e. the connections in the resultant temporal relatedness graph which, as explained in the previous section, depending on the local graph structure encode topic evolution, merging and splitting, and convergence and speciation.

**Fig. 8:**
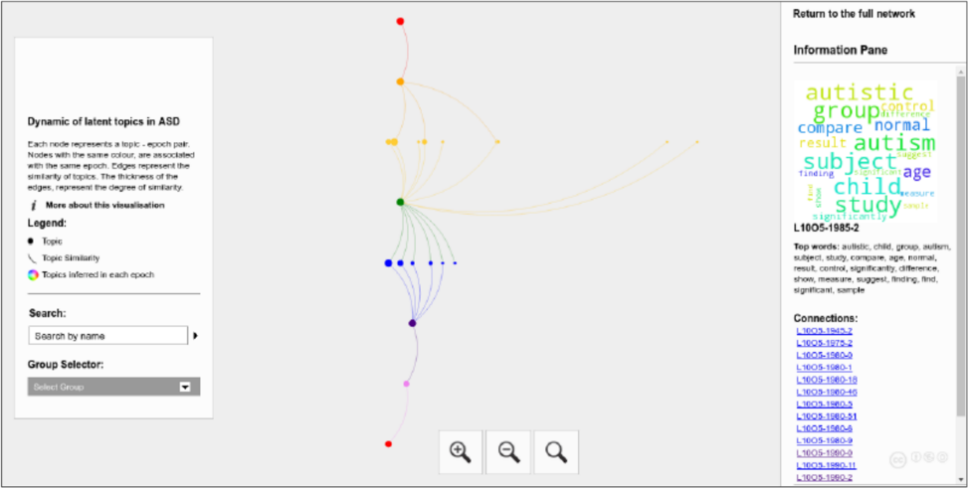
Illustration of some of the capabilities of the developed application: (*i*) the central part of the screen shows a selected topic and the strongest ancestral and descendent lineages, (*ii*) the search box on the left hand side can be used to enter multiple terms which are used to retrieve and rank topics, and (*iii*) on the right hand side, relevant information about the currently selected topic is summarized. Magnification tools are accessible from the bottom of the screen, whereas translation can be performed using a simple dragging motion

The application allows a range of powerful tasks to be performed quickly and in an intuitive manner. For example, the user can search for a given topic using keywords (and obtain a list of topics ranked by the degree to which they match the query), trace the origin of a specific topic backwards in time, or follow its development in the forward direction, examine word clouds associated with topics, display a range of statistical analyses, or navigate the temporal relatedness graph freely. Some of these capabilities are showcased in Fig. 8. In particular, the central part of the screen shows a selected topic and the strongest ancestral and descendent lineages. The search box on the left hand side can be used to enter multiple terms which are used to retrieve and rank topics by quality of fit to the query. Finally, on the right hand side, relevant information about the currently selected topic is summarized: its most popular terms are both visualized in the form of a colour coded word cloud, as well as listed in order in plain text underneath. Additional graph navigation options include magnification tools accessible from the bottom of the screen, whereas translation is readily performed by simply dragging the graph using a mouse or a touchpad.

## Conclusions

In this work, we presented a case for the importance of the use of advanced machine learning techniques in the analysis and interpretation of large longitudinal text corpora such as specialized medical literature. We described a novel framework based on non-parametric Bayesian techniques which is able to extract and track complex, semantically meaningful changes to the topic structure of a longitudinal document corpus. Moreover, this work is the first to describe and present a method for differentiating between two types of topic structure changes, namely topic splitting and what we termed topic speciation. Experiments on a large corpus of medical literature concerned with the metabolic syndrome was used to illustrate the performance of our method. Lastly, we developed a web application which allows users such as medical researchers to apply our method for their analysis.

The present work illuminates several promising avenues for future work. Firstly, considering the important difference between topic interaction phenomena such as topic splitting and speciation, and topic merging and convergence, we intend to focus on developing an information-theoretic framework which is capable of handing all of the aforementioned interactions in a unified manner. Such a framework would confer the additional benefit of removing the need for the free parameter necessitated by our method—the proportion of edges retained in our topic relatedness graph. The second direction of research we intend to pursue concerns the free parameters used to handle the temporal dimension. Although it is prima fasciae clear that the duration of the epoch length used to discretize time, as well as the degree of overlap between consecutive epochs, should be dependent on the dynamics of a particular longitudinal corpus (contrast for example the analysis of social media posts and the medical literature), currently, there are no principled methods for choosing the values of these parameters. Lastly, we believe that the nature of the problem at hand, that of temporal topic modelling, offers opportunities for a more objective manner of performance assessment, in contrast to the usual post hoc, qualitative approaches used in the existing literature on topic modelling.
